# Production and evaluation of novel functional cream cottage cheese fortified with bovine colostrum and probiotic bacteria

**DOI:** 10.1007/s13197-023-05910-0

**Published:** 2024-01-19

**Authors:** El-Sayed M. Abdeen, Ahmed M. Hamed, Hesham A. Ismail

**Affiliations:** 1 Animal and Poultry Breeding Department, Desert Research Center, Cairo, 11753 Egypt; 2 Dairy Science Department, Faculty of Agriculture, Cairo University, Giza, 12613 Egypt; 3 Dairy Science Department, Faculty of Agriculture, New Valley University, El-Kharga, 72511 Egypt

**Keywords:** Functional cream cottage cheese, Colostrum powder, Fatty acid profile, Color parameters, Texture analysis, Antioxidant activity

## Abstract

Nutrient-dense colostrum can be employed as a functional food ingredient. This work aimed to produce novel functional probiotic Cream cottage cheese (FPC) using probiotic (ABT) culture and bovine colostrum powder (BCP) at levels of 1, 2, and 3%. Physicochemical and functional properties (antioxidant activity, fatty acid profile, and antibacterial activity) were analyzed. The outcome revealed that hardness, cohesiveness, and gumminess were increased while springiness and chewiness were decreased for the treated cheeses. In FPC, medium-chain fatty acids were the predominant forms, followed by short- and long-chain fatty acids, polyunsaturated (PUFA), and small amounts of monounsaturated (MUFA). The antioxidant activity of all the cheese samples was significantly (*P* < 0.05) increased by increasing the quantity of colostrum powder and lengthening storage time. Color parameters were influenced by enrichment with BCP, whether in fresh or stored samples. With the addition of BCP, the growth of lactic acid bacteria and *Bifidobacteria* was enhanced, whereas that of pathogenic bacteria, mold and yeast, and coliform groups was inhibited. Cheeses fortified with 2% BCP had significantly higher score values than those in the other treatments. Therefore, it could be concluded that cottage cheese fortified with 2% BCP has high nutritional value and health benefits.

**Supplementary Information:**

The online version contains supplementary material available at 10.1007/s13197-023-05910-0.

## Introduction

Colostrum is the initial milk secreted from the mammals' mammary glands during the first days of calving and is substantially different from "mature" milk. Essential elements like proteins, lipids, vitamins, and minerals are distinctive in cow colostrum. Moreover, it has significant concentrations of bioactive substances such lactoferrin, oligosaccharides, immunoglobulins, and lysozyme (Kehoe et al. 2007) and anti-inflammatory, antioxidant, and immune-enhancing components that are not present in mature milk or are present in substantially lower concentrations (Bernabucci et al. 2013) Bovine colostrum has grown in popularity as a human diet in recent years because it is a great source of bioactive proteins, which are thought to prevent bacterial and viral infections, improve gastrointestinal health, and improve physical body condition (Houser et al. 2008). Furthermore, colostrum has numerous vitamins which have many roles in the body, including metabolism cofactors, oxygen transport, and antioxidants, and help the body use carbohydrates, protein, and fat. In addition, Bovine colostrum is known to be good sources of several minerals especially calcium and phosphorus (Pereira 2014). Several research has indicated that the average levels of certain essential minerals in colostrum are notably greater compared to mature bovine milk. Calcium is vital for the well-being of calves, as it supports their growth and ensures strong bones and teeth. Phosphorus is similarly significant for maintaining healthy metabolism and physiological processes, such as the formation of skeletal tissue, effective energy usage, protein production, and the transportation of fatty acids. (Haug, et al. 2007). As bovine colostrum had numerous advantages for human health, it is used in numerous interesting products, including Igazym lozenges, colostrum concentrate, spray-dried colostrum powder, colostrum capsules, and powder used to prepare different products in India like Azaco cheese, Kharwas, and Phuh (Nazir et al. 2018). As well as several dairy products like yogurt (Ayar et al. 2016; Arkan et al. 2022) and dahi (Das et al. 2013). Although leftover colostrum was previously regarded to be unmarketable, bioactive components in bovine colostrum have attracted interest in recent years as a potentially beneficial food ingredient for the future (O’Callaghan et al. 2020). Cottage cheese is fresh, soft, unripened, and produced through acid or enzymatically coagulated milk made from whole or skim milk (Ali et al. 2022). It is characterized by a high moisture content, a mildly acidic flavor, a smooth texture, and a short shelf life (Kosikowski and Mistry 1997). It contains the usual flavor of sour cream and can be flavored with fruit, spices, or herbs when eaten. Furthermore, probiotic populations could be utilized in the production of probiotic white cheese to shorten ripening time with higher levels of proteolysis and sensory acceptance (Kasmoglu et al. 2004). The synergistic health advantages of probiotics and colostrum can help to supply the food market and functional food industries with new products. Therefore, the objective of this research was to enhance cream cottage cheese production by adding varying amounts of colostrum powder and probiotic starter (ABT). To evaluate the quality of the cheese, several parameters such as physicochemical properties, fatty acid profile, color parameters, microbiological quality, and sensory characteristics were analyzed after one day of production and during a 21-day storage period at 5 °C. The texture of the cheese was only assessed in the freshly produced samples.

## Materials and methods

### Materials

Whole cow milk, which is separated into skim (0.4% fat) and cream (12% fat), used in this study, was obtained from the Dairy Unit, Faculty of Agriculture, New Valley University, Egypt. Bovine colostrum samples were collected within the first to fifth milking after parturition from a dairy farm in El-Kharga City, New Valley Governorate, Egypt. Immediately, the samples were transported to the laboratory and storage at –20 °C until use. Table salt was procured from the local market. Mesophilic homofermentative type O lactic culture consisting (*Lactococcus lactis* subsp. *cremoris* and *Lactococcus lactis* subsp. *lactis*) (10^8^ CFU/ml) and probiotic culture ABT YO-MIXÔ 205, consisting *Lactobacillus acidophilus*, Bifidobacterium spp., and *Streptococcus thermophiles* (10^9^ CFU/ml) were purchased in powder form (DANISCO EGYPT (Cairo, Egypt), and were both activated by being transferred three times in a row into 10% (w/v) reconstituted skim milk over 24 h at 30 °C and 37 °C, respectively.

## Methods

### Preparation of colostrum powder

The fresh colostrum was pasteurized following the method reported by Abd El-Fattah et al. (2014) at 60 °C for 60 min, which they reported that it had no effect on the IgG activity and viscosity of colostrum and was very effective against *S. aureus*, Salmonella spp., and *E. coli.* Then, freeze-drying for colostrum was done in the laboratory of the National Research Center, Cairo, Egypt. Colostrum samples were immediately placed in specially designed glass bottles for freeze-drying and stored at − 80 °C for 24 h. The frozen glass bottles were then freeze-dried at − 50 °C for 48 h under vacuum (0.05 mbar) using Labconco freeze dryer, Console, 12L, -50 °C, Stoppering Tray Dryer, Free Zone, 240 V, Catalog No. 7754030, Serial No. 100931482 D, U.S.A.

### Cream fermentation

The cream fermentation preparation was obtained by mixing the previously obtained cream (12% fat). The cream was pasteurized at 85 °C for 15 s, put in stainless steel vessels, and inoculated with probiotic culture ABT at 37 °C for 3–5 h to obtain an initial concentration of 5 × 10^7^ CFU/g of cream. The fermented cream was then refrigerated at 4 °C until it was time to make cottage cheese (about 12 h).

### Preparation of cream cottage cheese

Standardized milk (0.4% fat, 3.95% protein, 4.81% lactose, and 0.73% ash) was pasteurized at 72 °C for 15–20 s and cooled to 35 °C before adding 2% mesophilic culture. The mixture was maintained at the same temperature for approximately 12 h for fermentation until the pH dropped to 4.7 and complete coagulation occurred. Curds were divided into smaller cubes by cutting them both horizontally and vertically, and they were then given 10 min to mature. For 2 h, the temperature of the curds was gradually raised to 55–60 °C (scalding process). Curds were washed once with water at 50 °C to remove the lactose from the curd and again with water at 10 °C. Colostrum powder was added to drained curd cheese at ratios of 1%, 2%, and 3%. After mixing it with fermented cream and blended for 30–40 min at 12 °C. Then, sodium chloride was added to mixur and mixed well to give a product with an approximate composition of 4% fat and 1% NaCl, previously coded (T1, T2, and T3, respectively). Curd without colostrum powder was kept as the control (C). The cream cottage cheeses were packaged in clean polyethylene bags and stored at 5 °C for 21 days. Physicochemical properties, fatty acid profile, color parameters, microbiological quality, and organoleptic characteristics were carried out at 1, 7, 14, and 21-day intervals, and texture features were determined only in fresh samples. This procedure was used three times to make cream cottage cheese to test the repeatability of the finished product's features.

## Analysis

### Physicochemical analysis

The chemical composition of colostrum powder and cheese was determined following the standard methods of AOAC (2000): total solids by drying at 105 °C; total proteins by the Kjeldahl method (factor = 6.38); fat by the Gerber method; ash by incineration at 550 °C; lactose was calculated by the difference of the total contents (protein, fat, and ash) from the total solids. pH value by using a digital pH meter (model Adwa 1030, Romania). Total titratable acidity was determined by titration with 0.1 N NaOH using drops of 1% (w/w) phenolphthalein as an indicator. However, the lipid content of colostrum powder, measured by Soxhlet extraction with petroleum ether and vitamin A, was determined according to Brubacher et al. (1985). Minerals were analyzed following the standard methods of AOAC (2000): calcium (Ca), phosphorus (P), magnesium (Mg), iron (Fe), zinc (Zn), and copper (Cu) content were determined using a Perkin-Elmer atomic absorption spectrophotometer, while sodium (Na) and potassium (K) contents were measured using a flame photometer (CORNING 400, serial no. 4889, UK).

### Fatty acids analysis

The analysis of fatty acids was conducted at the central laboratory of the Desert Research Center, Ministry of Agriculture and Land Reclamation, Cairo, Egypt. Gas chromatography (GC) was utilized for this analysis, following the guidelines outlined in the AOAC 963.22 method (AOAC 2000).The sample was dissolved in 1 ml of hexane and then in 10 ml of KOH (2N) in methanol (11.2 g in 100 ml). The tube was vortexed for 30 s. Gas chromatography was used to conduct FAME analysis, utilizing a Thermo (Italy) focus model gas chromatograph equipped with a flame-ionization detector (GC-FID) and a split–splitless injector port. The FAMEs were separated chromatographically using a CP 7419 FAME capillary column Supelco DB-Wax (Carbowax) with dimensions of 30 m long and 0.25 mm with a phase thickness of 0.25 μm. Helium was utilized as the carrier gas at a flow rate of 40 mL/min. The samples were injected using the inlet in a split mode, with one μL injected. The head pressure was set at 2 psi, and the split vent flow was 7 mL/min. The injector temperature was maintained at 250 °C. The column flow rate was 40 mL/min at 2 psi, and the column temperature was kept at 50 to 200 °C with a rate of 10 °C/s, held at 260 °C for 80 min. The fatty acids were identified using retention times obtained from FAME standards (Sigma Company, St. Louis, MO) and were expressed as g/100 g of total fatty acids, calculated with peak areas corrected by factors according to AOAC 963.22 method (AOAC 2000).

### Antioxidant activity (DPPH) radical scavenging assay

The ability of the antioxidant to scavenge stable 1,1-diphenyl-2-picrylhydrazyl (DPPH) radicals was evaluated spectrophotometrically at 517 nm using a UV/Vis spectrophotometer (Jenway, Staffordshire, ST15 OSA, UK), concerning the radical-scavenging ability of the reference substance, 6-hydroxy-2,5,7,8-tetramethylchromane2-carboxylic acid (Trolox). The absorbance decrease is a result of the radical-scavenging ability of the substance. Results were presented as millimoles of Trolox equivalents (TE) per liter, based on a standard for Trolox.

### Color parameters

Color parameters of the samples were estimated by using a Minolta colour reader (Minolta Camera Co., Ltd., Osaka, Japan). In this method, the L* value, which represents lightness, ranges from 0 (black) to 100 (white), the a* value, which represents greenness, from − 100 to + 100 (redness), and the b* value, which represents blueness, from − 100 to + 100 (yellowness). The hue angle (H*), chroma (C*), yellowing index (YI), and browning index (BI) were calculated with the following equations according to (Milovanovic et al. 2020). Hue angle (h°) refers to the degree of the dominant spectral component, such as red, green, and blue, and ranges from 0 to 360°. An angle of 0° or 360° represents red Hue, while angles 90°, 180°, and 270° represent yellow, green, and blue Hue, respectively. Combining a* and b*, h° better represents the color; it is calculated based on formula (1)

1 $$H* = {\text{arctan }}\left( {b*/a*} \right)$$

The chroma (C*) represents the vividness or saturation of a color and is defined as follows: (2):

2 $$C* \, = \, \left( {a*^{{2}} + b*^{{2}} } \right)^{{{1}/{2}}}$$

The yellowing index (YI) is used as a color measurement related to browning index

3 $${\text{YI}} = {142}.{86} \times {\text{b}}^{ * } /{\text{L}} *$$

The browning index (BI) is defined as brown color purity and is one of the most common indicators of browning in food products containing sugar. The BI is calculated using the following expression

4 $${\text{BI}} = ({1}00 \times ({\text{x}} - 0.{31})) \, /0.{17}$$

5 $${\text{Where}} {\text{X}} = ({\text{a}}* + {1}.{\text{75L}})/({5}.{\text{645L}} + {\text{a}}* - {3}.0{\text{12b}}*)$$

### Texture profile analysis (TPA)

Texture profile analysis was performed using a TMS-Pro Texture Analyzer (Stable Micro System, USA) equipped with a 25 N force load cell. Each sample (6.5 cm in diameter and 5 cm in height) was squeezed twice using a 25 mm diameter cone probe. The optimized test conditions were a 60 mm back-off distance to the sample, 35% deformation ratio, 60 mm minG1 test speed, and 0.15 N trigger force using Cone Probe of 25 mm diameter. TL-Pro software was used to assess the hardness, adhesiveness, cohesiveness, springiness, gumminess, and chewiness of cheese samples. All measurements were analyzed at 20 °C.

### Microbiological analysis

Lactic acid bacteria counts and Bifidobacteria were enumerated by the method of Tharmaraj and Shah (2003). The resulting Cream cottage cheese samples were tested for total bacterial, mold, and yeast counts, as well as coliform groups according to Harrigan (2014).

### Organoleptic characteristics

Cream cottage cheese samples were evaluated by 10 panelists, all staff members of the Dairy Science Department, Faculty of Agriculture, New Valley University. It is scored for flavor (1–10 points), body and texture (1–5 points), and appearance (1–5 points), where 1 = "extremely undesirable" and 5 = "excellent," while the overall grade (1–20) is determined. The prepared cream cheese samples were cut, placed on white plates, and presented to the panelists randomly. Water was provided for mouth washing between samples. This research was conducted in cooperation between the Dairy Science Department, Faculty of Agriculture, New Valley University, the Animal and Poultry Breeding Department, Desert Research Center, Cairo, and the Dairy Science Department, Faculty of Agriculture, Cairo University, Egypt.

### Statistical analysis

Measurements were carried out in three replicates, whereas the sensory evaluation was carried out in ten replicates, and the data were presented as mean ± standard deviation (SD) and analyzed statistically using the SPSS 16.0 software package with one-way analysis of variance (ANOVA) and Duncan's Multiple Range Test at *P* < 0.05.

## Results and discussion

### Physicochemical properties of colostrum powder used in the manufacturing of cream cottage cheese

The physicochemical properties of colostrum powder are presented in Table 1. The percentages of total solids, total protein, fat, lactose, and ash in bovine colostrum powder were 95.39, 40.75, 29.11, 16.33, and 9.2%, respectively. The results agree with those of previous studies by Sert et al. (2015), who found that colostrum powder comprises total solids; total protein, fat, ash, and lactose were 96.0, 32.4, 36.1, 11.4, and 16.1, respectively. The level of vitamin A (Table 1) was 16.87 IU/g of colostrum powder. Bovine colostrum contains high amounts of water- and fat-soluble vitamins, which are essential for human health (McGrath et al. 2016). As seen in Table 1, bovine colostrum is a rich source of many minerals. Kehoe et al. (2007) reported that the mean calcium and phosphate concentrations in colostrum were, respectively, approximately 4- and 5-times greater than those in milk.

**Table 1:** Physicochemical composition and minerals contents of bovine colostrum powder used for Cream cottage cheese manufacture

Physic-chemical properties	Value	Minerals contents	Value
Total solids (%)	95.39 ± 1.01	Calcium (mg/100 g)	1866.89 ± 77.48
SNF (%)	66.28 ± 0.81	Iron(mg/100 g)	24.60 ± 0.72
Protein (%)	40.75 ± 0.89	Magnesium(mg/100 g)	237.67 ± 4.16
Fat (%)	29.11 ± 0.95	Phosphorous(mg/100 g)	1536.00 ± 66.78
Lactose (%)	16.33 ± 0.155	Potassium(mg/100 g)	1032.86 ± 16.53
Ash (%)	9.197 ± 0.519	Sodium(mg/100 g)	411.22 ± 3.62
Acidity (%)	0.187 ± 0.025	Zinc(mg/100 g)	14.61 ± 0.64
pH value	6.55 ± 0.105	Copper(mg /100 g)	1.12 ± 0.01
Vitamin (A) (IU /g)	16.87 ± 0.21	Manganese(mg /100 g)	2.75 ± 0.12

Means (three different determinations) ± standard deviation (SD)

### Fatty acid profile of colostrum powder used in the manufacturing of Cream cottage cheese

The profile of fatty acids in bovine colostrum powder is presented in Fig. S1. It can be observed that the content of short-chain fatty acids (C4-C8) in colostrum powder was lower than that of medium-chain fatty acids (C10-C14) and long-chain fatty acids (C16-C20). These results are in good accordance with those reported by Laakso et al. (1996). However, short-chain fatty acids represent 8% of the proportion of saturated acids, which is a significant proportion. According to those studies, short-chain fatty acids regulate lipid metabolism and intestinal flora by modifying intestinal pH, which has a significant impact on the health of infants. The results also showed an increase in the amounts of myristic and palmitic acids as well as the unsaturated fatty acids, especially C14:1, C18:1, and C20:4. Due to the cows' negative energy balance during parturition, which causes the mobilization of adipose tissue that is then integrated into milk fat, colostrum contains high quantities of long-chain fatty acids. The fats present in colostrum, specifically palmitic, palmitoleic, and myristic acids, differ from those in mature milk (Arslan et al. 2021). High quantities of long-chain fatty acids also prevent the de novo synthesis of short-chain fatty acids. Oleic acid (C18:1) is an important source of energy for breastfed infants. Conjugated linoleic acid is one of the fatty acids found in colostrum that helps to prevent cancer. Studying the fatty acid composition of bovine colostrum revealed that it may serve as a functional dietary alternative for people since it contains fewer saturated fatty acids than milk and more unsaturated fatty acids, including linoleic-conjugated acid, which is good for human health (Mašek et al. 2014) and reduces cholesterolemia. Polyunsaturated fatty acids are crucial for the formation of cell membranes, are precursors in the production of prostaglandins, thromboxanes, and leukotrienes (ω6 family), and support healthy neurological and visual development.

### Physicochemical analysis of cream cottage cheese

Colostrum has attracted more interest due to its bioactive makeup as a potential dietary ingredient with health benefits. The physical and chemical properties of cream cottage cheese samples fortified with colostrum powder during storage at 5 °C for 21 days are given in Table 2. The addition of 1, 2, and 3% BCP significantly increased the total solids, protein, ash, and carbohydrate contents of the treatment FPC compared with the control. These results conformed to those given by Das and Seth (2017), who found that adding 2% bovine colostrum whey powder to curd caused a significant increase in total protein compared to the control curd. This could be because colostrum contains a higher amount of all these constituents as compared to mature milk. Therefore, combining cheese with colostrum can increase the cheese's quality, especially in physical characteristics like texture (Poonia and Dabur 2015). During storage, total solids, protein, and ash contents increased. This may be due to the evaporation of some moisture and the formation of a gel by the use of chemically bound water, which also cause an increase in the total solid content of cottage cheese during the storage period. Furthermore, the decrease in moisture content of cottage cheese can affect the biochemical and enzymatic activities that are always developed during storage and take place in the presence of water (Ali et al. 2022). Fat contents in the control were higher than those of other cottage cheese treatments, and all cheese samples decreased from the first week to the end of the storage period. These results were in agreement with the study by Das and Seth (2017). The use of colostrum powder resulted in a significant increase in acidity and reduction in pH of the cream cottage cheese treatments compared to the control (Table 2). This may be due to colostrum powder having more lactose than the control, as shown in the treatments compared to the control. The presence of lactic acid bacteria decomposes more biochemical products like lactose and ferments them into lactic acid, which reduces the pH and increases the titratable acidity of cottage cheese (Kosikowski and Mistry 1997). As a result, the acidification rate increased as the amount of colostrum added increased. During storage, the pH of cream cottage cheese decreases while the titratable acidity increases in a correlated manner. In general, it was noted that the pH values of all samples of cheese treatment showed a significant (*P* < 0.05) decrease with an advanced storage period until the 21st day as a result of post-fermentation of lactose to lactic acid. The pH of cheese is one of the most crucial factors that influence how probiotic bacteria grow in it. As a result, because the pH of the cheese matrix is closer to its ideal range of 4.8–5.6, probiotic bacteria can grow here more readily.

**Table 2:** Effect of enrichment with colostrum powder on physicochemical properties of the resultant Cream cottage cheese during storage

Cream cottage cheese	Storage period (day)			
	1	7	14	21
TS				
*C*	24.50 ± 0.98 ^cA^	24.64 ± 0.35 ^cA^	24.87 ± 0.25 ^dA^	25.17 ± 0.77 ^dA^
*T1*	25.59 ± 0.45 ^bA^	25.77 ± 0.41 ^bA^	25.81 ± 0.23 ^cA^	26.24 ± 0.28 ^cA^
*T2*	26.52 ± 0.31 ^abB^	26.91 ± 0.22 ^aAB^	26.98 ± 0.22 ^bAB^	27.34 ± 0.27 ^bA^
*T3*	27.13 ± 0.21 ^aC^	27.47 ± 0.12 ^aBC^	27.58 ± 0.24 ^aB^	28.21 ± 0.28 ^aA^
Protein				
*C*	16.41 ± 0.40 ^cB^	16.63 ± 0.48 ^cAB^	17.02 ± 0.31 ^dAB^	17.42 ± 0.42 ^dA^
*T1*	17.31 ± 0.20 ^bC^	17.65 ± 0.26 ^bBC^	17.86 ± 0.21 ^cAB^	18.32 ± 0.31 ^cA^
*T2*	18.02 ± 0.19 ^aC^	18.45 ± 0.37 ^aBC^	18.66 ± 0.21 ^bB^	19.23 ± 0.27 ^bA^
*T3*	18.53 ± 0.36 ^aC^	18.84 ± 0.08 ^aBC^	19.18 ± 0.21 ^aB^	19.97 ± 0.11 ^aA^
Fat				
*C*	4.00 ± 0.17 ^aA^	3.97 ± 0.12 ^aAB^	3.83 ± 0.12 ^aAB^	3.73 ± 0.06 ^aB^
*T1*	4.00 ± 0.17 ^aA^	3.93 ± 0.15 ^aA^	3.77 ± 0.06 ^abAB^	3.67 ± 0.06 ^abB^
*T2*	4.00 ± 0.17 ^aA^	3.90 ± 0.10 ^aA^	3.67 ± 0.10 ^bB^	3.57 ± 0.06 ^bcB^
*T3*	4.00 ± 0.10 ^aA^	3.87 ± 0.06 ^aA^	3.63 ± 0.06 ^bB^	3.50 ± 0.10 ^cB^
Carbohydrates				
*C*	2.32 ± 0.04 ^cA^	2.22 ± 0.05 ^cAB^	2.12 ± 0.03 ^cBC^	2.07 ± 0.09 ^cC^
*T1*	2.44 ± 0.04 ^bA^	2.32 ± 0.04 ^bB^	2.21 ± 0.04 ^bC^	2.14 ± 0.04 ^bcC^
*T2*	2.59 ± 0.07 ^aA^	2.57 ± 0.04 ^aA^	2.47 ± 0.05 ^aB^	2.23 ± 0.02 ^bC^
*T3*	2.61 ± 0.05 ^aA^	2.60 ± 0.08 ^aA^	2.51 ± 0.03 ^aA^	2.34 ± 0.04 ^aB^
Ash				
*C*	1.77 ± 0.13 ^bA^	1.82 ± 0.08 ^cA^	1.90 ± 0.10 ^bA^	1.95 ± 0.07 ^cA^
*T1*	1.84 ± 0.12 ^bB^	1.87 ± 0.06 ^cB^	1.97 ± 0.07 ^bAB^	2.11 ± 0.04 ^bA^
*T2*	1.91 ± 0.06 ^abD^	1.99 ± 0.05 ^bC^	2.18 ± 0.01 ^aB^	2.31 ± 0.03 ^aA^
*T3*	2.07 ± 0.14 ^aB^	2.16 ± 0.07 ^aB^	2.26 ± 0.08 ^aAB^	2.40 ± 0.09 ^aA^
pH				
*C*	4.63 ± 0.12 ^aA^	4.55 ± 0.12 ^aB^	4.47 ± 0.05 ^aB^	4.40 ± 0.06 ^aC^
*T1*	4.52 ± 0.13 ^bA^	4.48 ± 0.10 ^bAB^	4.41 ± 0.07 ^bBC^	4.35 ± 0.05 ^bC^
*T2*	4.46 ± 0.09 ^cA^	4.41 ± 0.17 ^cB^	4.34 ± 0.05 ^cBC^	4.27 ± 0.09 ^cC^
*T3*	4.38 ± 0.15 ^cA^	4.31 ± 0.02 ^cAB^	4.27 ± 0.05 ^cB^	4.21 ± 0.11 ^cC^
Acidity				
*C*	0.74 ± 0.02 ^bA^	0.77 ± 0.12 ^aA^	0.79 ± 0.02 ^bA^	0.82 ± 0.09 ^bA^
*T1*	0.76 ± 0.03 ^bC^	0.79 ± 0.02 ^aBC^	0.82 ± 0.02 ^bAB^	0.85 ± 0.04 ^abA^
*T2*	0.78 ± 0.03 ^abB^	0.81 ± 0.04 ^aAB^	0.85 ± 0.06 ^abAB^	0.88 ± 0.03 ^abA^
*T3*	0.82 ± 0.03 ^aB^	0.85 ± 0.04 ^aAB^	0.91 ± 0.03 ^aAB^	0.95 ± 0.06 ^aA^

Mean (± SD) with small letters (abc) indicate significant differences (*P* < 0.05) among Cream cottage cheese samples enriched with different colostrum powder levels. Mean (± SD) with capital letters (ABC) indicate significant differences *P* < 0.05) among Cream cottage cheese samples during storage,where: C, control Cream cottage cheese; T1, Cream cottage cheese with 1% colostrum powder; T2, Cream cottage cheese with 2% colostrum powder; T3, Cream cottage cheese with 3% colostrum powder

### Fatty acids profile of cream cottage cheese

Data presented in Table 3 demonstrated the effect of fortification by colostrum powder on the fatty acid contents of cream cottage cheese during storage at 5 °C for 21 days. As verified in previous studies (Mašek et al. 2014), medium-chain fatty acids were the predominant form, followed by short-, long-chain fatty acids, polyunsaturated fatty acids (PUFA), and small amounts of monounsaturated fatty acids (MUFA). This was similar to the current study. The results show that short-chain fatty acids were increased by adding colostrum powder to the curd, and the highest content was from the T3 treatment, compared to other trail cheeses. As well, through the storage period, the short-chain fatty acids increased up to 21 days. The amount of short-chain fatty acids accumulated during ripening could be interpreted as an overall measure of lipolysis and protein breakdown, which improved the flavor of the finished product. The medium-chain fatty acids in FPC samples had a lower content compared to the control and declined throughout the storage periods until the end of storage. The percentage of long-chain fatty acids increased with the addition of colostrum powder but was seen to significantly (*P* < 0.05) drop after 14 days until the end of storage. At the same time, the proportion of unsaturated fatty acids (monounsaturated and polyunsaturated) increased with the addition of colostrum powder, differences were significant (*P* < 0.05) in polyunsaturated fatty acids. Storage was also found to exert a negative effect on the unsaturated fatty acid content of the cheese, as it declined steadily from the first week to the third week. Because unsaturated fatty acids are particularly susceptible to lipid oxidation, the proportions of different fatty acid groups may have changed. The results in the present study were in agreement with those data obtained by Laskaridis et al. (2013), they found an increase in the content of unsaturated fatty acids in the fat of Feta cheese made from sheep milk during the initial period of storage, with a subsequent substantial decline in the content of MUFA and PUFA after about 40 days. Bomba et al. (2019) analyzed the fatty acid composition of cottage cheese after 4 weeks of storage and found that there is a slight decrease in stearic (C18:00) and oleic acid (C18:1 Δc). Fletouris et al. (2014) studied the changes in the fatty acid composition of Graviera Agraphon cheese during storage for 60 days. After 30 days, they observed a significant (*P* < 0.05) increase in the level of SFA, after which it remained stable, while the total concentration of MUFA and PUFA declined at 30 days of storage and then remained consistent.

**Table 3:** Effect of enrichment with colostrum powder on fatty acid profile of the resultant Cream cottage cheese during storage

Fatty acid		Storage period (day)	Cream cottage cheese			
			C	T1	T2	T3
Short- chain fatty acids (SCSFA C4:0-C8:0)	Saturated fatty acids	1	14.12 ± 0.04 ^bD^	14.57 ± 0.10 ^aC^	14.59 ± 0.12 ^aD^	14.64 ± 0.07 ^aD^
		7	14.46 ± 0.11 ^cC^	14.66 ± 0.23 ^bcC^	14.82 ± 0.06 ^abC^	15.08 ± 0.17 ^aC^
		14	15.23 ± 0.12 ^dB^	15.56 ± 0.13 ^cB^	15.80 ± 0.08 ^bB^	16.07 ± 0.06 ^aB^
		21	16.16 ± 0.11 ^dA^	16.36 ± 0.10 ^cA^	16.63 ± 0.06 ^bA^	16.87 ± 0.10 ^aA^
Medium- chain fatty acids (MCSFA C10:0-C14:0)		1	63.75 ± 0.42 ^abA^	64.56 ± 0.71 ^aA^	62.59 ± 0.92 ^bcA^	61.80 ± 1.01 ^cA^
		7	63.50 ± 0.65 ^aA^	62.90 ± 0.62 ^abB^	62.10 ± 0.49 ^bcAB^	61.48 ± 0.45 ^cA^
		14	62.20 ± 0.39 ^aB^	62.09 ± 0.44 ^aB^	61.71 ± 0.23 ^aAB^	60.27 ± 0.12 ^bB^
		21	61.48 ± 0.46 ^aB^	61.02 ± 0.15 ^aC^	60.95 ± 0.68 ^aB^	60.67 ± 0.39 ^aAB^
long -chain fatty acids (LCSFAC 16:0-C22)		1	10.09 ± 0.16 ^bB^	11.11 ± 0.23 ^bC^	11.57 ± 0.32 ^abC^	12.57 ± 0.26 ^aA^
		7	10.40 ± 0.23 ^dAB^	11.57 ± 0.19 ^cB^	12.13 ± 0.18 ^bB^	12.79 ± 0.15 ^aA^
		14	10.53 ± 0.13 ^bA^	11.73 ± 0.09 ^aA^	12.90 ± 0.15 ^aA^	12.93 ± 0.14 ^aA^
		21	9.38 ± 0.25 ^dC^	10.01 ± 0.14 ^cD^	10.96 ± 0.24 ^bD^	11.40 ± 0.17 ^aB^
Monounsaturated fatty acids (MUFA)	Unsaturated fatty acids	1	2.82 ± 0.10 ^bA^	2.84 ± 0.11 ^bAB^	2.95 ± 0.09 ^abA^	3.10 ± 0.12 ^aA^
		7	2.62 ± 0.12 ^bB^	2.94 ± 0.08 ^aA^	3.03 ± 0.12 ^aA^	3.08 ± 0.06 ^aA^
		14	2.26 ± 0.08 ^bC^	2.91 ± 0.18 ^aA^	2.98 ± 0.11 ^aA^	3.03 ± 0.10 ^aA^
		21	2.09 ± 0.10 ^bC^	2.65 ± 0.10 ^aB^	2.72 ± 0.13 ^aB^	2.81 ± 0.12 ^aB^
polyunsaturated fatty acids (PUFA)		1	6.22 ± 0.25 ^bA^	6.43 ± 0.27 ^bB^	6.52 ± 0.21 ^bC^	7.89 ± 0.09 ^aB^
		7	6.02 ± 0.14 ^dB^	6.94 ± 0.07 ^cA^	7.92 ± 0.08 ^bA^	8.18 ± 0.11 ^aA^
		14	5.95 ± 0.09 ^dAB^	6.41 ± 0.13 ^cB^	6.98 ± 0.13 ^bB^	7.89 ± 0.12 ^aB^
		21	5.73 ± 0.07 ^dB^	6.40 ± 0.15 ^cB^	6.99 ± 0.14 ^bB^	7.25 ± 0.08 ^aC^

Mean (± SD) with small letters (abc) indicate significant differences (*P* < 0.05) among Cream cottage cheese samples enriched with different colostrum powder levels. Mean (± SD) with capital letters (ABC) indicate significant differences *P* < 0.05) among Cream cottage cheese samples during storage,where: C, control Cream cottage cheese; T1, Cream cottage cheese with 1% colostrum powder; T2, Cream cottage cheese with 2% colostrum powder; T3, Cream cottage cheese with 3% colostrum powder

### Antioxidant activity

Table 4 shows the DPPH radical scavenging activity of Cream cottage fortified with bovine colostrum powder during storage at 5 °C for 21 days. The percentage of inhibition of DPPH radicals in all the cheese samples increased with increasing BCP concentration and storage time. These findings are consistent with those of Arkan et al. (2022), who discovered that yogurt made from combined milk and colostrum (20:80%) can increase antioxidant activity. There were significant differences (*P* < 0.05) in DPPH values between different treatments (0.149, 0.117, 0.114, and 0.101 mmol TE/L for fresh samples of T3, T2, and T1, respectively) and the end of the storage period (0.292, 0.230, 0.222, and 0.198 mmol TE/L for 21-day samples of T3, T2, and T1, respectively).

**Table 4:** Effect of enrichment with colostrum powder on antioxidant activity and color properties of the resultant Cream cottage cheese during storage

Cream Cottage cheese		Storage period (day)			
		1	7	14	21
Antioxidant activity					
*C*	DPPH (mmol TE/L)	0.103 ± 0.011 ^bC^	0.126 ± 0.012 ^bC^	0.158 ± 0.016 ^cB^	0.198 ± 0.020 ^cA^
*T1*		0.114 ± 0.011 ^bC^	0.142 ± 0.014 ^bC^	0.178 ± 0.018 ^bcB^	0.222 ± 0.022 ^bcA^
*T2*		0.117 ± 0.012 ^bC^	0.147 ± 0.015 ^bC^	0.204 ± 0.020 ^abB^	0.260 ± 0.026 ^abA^
*T3*		0.149 ± 0.015 ^aC^	0.186 ± 0.018 ^bC^	0.233 ± 0.023 ^aB^	0.292 ± 0.029 ^aA^
Color parameters					
*C*	L*	62.45 ± 0.09 ^aA^	56.74 ± 0.35 ^aB^	53.66 ± 0.38 ^aC^	52.11 ± 0.17 ^aD^
*T1*		61.9 ± 60.19 ^bA^	53.58 ± 0.30 ^bB^	52.82 ± 0.91 ^bC^	51.91 ± 0.28 ^aD^
*T2*		60.69 ± 0.35 ^cA^	52.37 ± 0.89 ^cB^	51.46 ± 0.45 ^cB^	50.43 ± 0.40 ^bC^
*T3*		58.19 ± 0.15 ^dA^	51.48 ± 0.58 ^dB^	50.61 ± 0.31 ^dC^	49.75 ± 0.28 ^cD^
*C*	a*	− 3.54 ± 0.19 ^cB^	− 3.35 ± 0.23 ^cB^	− 1.76 ± 0.17 ^bA^	− 1.54 ± 0.11 ^bA^
*T1*		− 3.10 ± 0.13 ^bB^	− 2.84 ± 0.11 ^bB^	− 1.64 ± 0.28 ^abA^	− 1.40 ± 0.20 ^abA^
*T2*		− 2.81 ± 0.17 ^bC^	− 2.33 ± 0.14 ^aB^	− 1.44 ± 0.19 ^abA^	− 1.20 ± 0.14 ^aA^
*T3*		− 2.32 ± 0.20 ^aB^	− 2.07 ± 0.14 ^aB^	− 1.30 ± 0.15 ^aA^	− 1.10 ± 0.14 ^aA^
*C*	b*	10.53 ± 0.43 ^dD^	11.12 ± 0.13 ^cC^	13.23 ± 0.07 ^cB^	13.78 ± 0.13 ^cA^
*T1*		11.12 ± 0.16 ^cD^	12.35 ± 0.10 ^bC^	14.11 ± 0.11 ^bB^	14.57 ± 0.14 ^bA^
*T2*		12.34 ± 0.26 ^bC^	13.56 ± 0.27 ^aB^	14.50 ± 0.27 ^aA^	14.91 ± 0.12 ^aA^
*T3*		13.50 ± 0.27 ^aC^	13.74 ± 0.07 ^aC^	14.64 ± 0.13 ^aB^	15.00 ± 0.16 ^aA^
*C*	Hug (H)	− 1.25 ± 0.02 ^aA^	− 1.28 ± 0.02 ^aB^	− 1.44 ± 0.01 ^aC^	− 1.46 ± 0.01 ^aC^
*T1*		− 1.30 ± 0.01 ^bA^	− 1.34 ± 0.01 ^bB^	− 1.46 ± 0.02 ^abC^	− 1.48 ± 0.01 ^abC^
*T2*		− 1.35 ± 0.02 ^cA^	− 1.40 ± 0.01 ^cB^	− 1.47 ± 0.01 ^bC^	− 1.49 ± 0.01 ^bcC^
*T3*		− 1.40 ± 0.02 ^dA^	− 1.42 ± 0.01 ^cA^	− 1.48 ± 0.01 ^bB^	− 1.50 ± 0.01 ^cB^
*C*	Chroma (C)	61.80 ± 4.44 ^cD^	67.50 ± 1.88 ^cC^	89.11 ± 0.67 ^cB^	96.19 ± 1.76 ^cA^
*T1*		66.66 ± 1.38 ^cD^	80.30 ± 1.09 ^bC^	100.87 ± 1.29 ^bB^	107.09 ± 2.09 ^bA^
*T2*		80.16 ± 2.77 ^bC^	94.63 ± 3.31 ^aB^	106.25 ± 3.80 ^aA^	111.89 ± 2.02 ^aA^
*T3*		93.85 ± 3.23 ^aC^	96.55 ± 1.09 ^aC^	107.98 ± 1.69 ^aB^	113.12 ± 2.28 ^aA^
*C*	Yellowing index (YI)	24.10 ± 0.99 ^dD^	28.01 ± 0.35 ^cC^	35.24 ± 0.40 dB	37.79 ± 0.45 ^dA^
*T1*		25.64 ± 0.33 ^cD^	32.93 ± 0.46 ^bC^	38.16 ± 0.13 ^cB^	40.09 ± 0.43 ^cA^
*T2*		29.06 ± 0.66 ^bD^	36.99 ± 1.10 ^aC^	40.26 ± 0.56 ^bB^	42.24 ± 0.49 ^bA^
*T3*		33.14 ± 0.70 ^aD^	38.13 ± 0.28 ^aC^	41.32 ± 0.50 ^aB^	43.07 ± 0.25 ^aA^
*C*	BI	13.72 ± 0.90 ^dD^	16.76 ± 0.23 ^dC^	25.10 ± 0.58 ^dB^	27.66 ± 0.40 ^dA^
*T1*		15.49 ± 0.44 ^cD^	21.46 ± 0.49 ^cC^	27.89 ± 0.48 ^cB^	30.01 ± 0.58 ^cA^
*T2*		18.64 ± 0.74 ^bD^	25.77 ± 1.07 ^bC^	30.08 ± 0.72 ^bB^	32.29 ± 0.34 ^bA^
*T3*		22.68 ± 0.88 ^aD^	27.14 ± 0.44 ^aC^	31.26 ± 0.64 ^aB^	33.22 ± 0.43 ^aA^

Mean (± SD) with small letters (abc) indicate significant differences (*P* < 0.05) among Cream cottage cheese samples enriched with different colostrum powder levels. Mean (± SD) with capital letters (ABC) indicate significant differences *P* < 0.05) among Cream cottage cheese samples during storage,where: C, control Cream cottage cheese; T1, Cream cottage cheese with 1% colostrum powder; T2, Cream cottage cheese with 2% colostrum powder; T3, Cream cottage cheese with 3% colostrum powder

### Color parameter

Food color is a significant factor in whether a consumer would accept or reject the final product and is thought to be a sign of changes in the food's nutritional value and organoleptic qualities that occur throughout storage. Table 4 shows the color characteristics of Cream Cottage fortified with Bovine Colostrum Powder during storage at 5 °C for 21 days. It could be noticed that using BCP in cream cottage cheese formulations affected the color values, and significant differences were noticed between the color attributes of the treatment cheese and those of the control samples. Lightness (L* values) was reduced by increasing the colostrum powder ratio in cheese formulas. Control cheese (C) had the highest L* value (62.45), while treatment (T3) had the lowest one (58.19). The lower L* value for treatment cheeses could be attributed to the yellow color of colostrum, which increased the yellow color of all FPC samples more than the control. Furthermore, the lightness (L* value) of all cheese samples was reduced during the storage time. This could be attributed to the increase in acidity and proteolysis that occurs during the storage period and the transformation of casein into a more soluble state, which may lead to a decrease in whiteness. The a* values (redness) of cheese samples were significantly increased (*P* < 0.05) when higher levels of colostrum powder were added. Similarly, Ayar et al. (2016) found that a significant increase in the a* values resulted from the addition of colostrum to yogurt. Additionally, the average redness (a*) of all cheeses increased with an increased storage period (Table 4). These results may be due to the a* values of cheese being influenced by microbiological, biochemical, chemical, and physical changes that happen during processing and storage. The b* value showed a significant increase when the proportion of colostrum was increased. The yellow color in colostrum hints at beta-carotene pigment, so the cheese produced is dark yellow, which affects the lightness (L*) of the cheese. The outcomes are consistent with those established by Ayar et al. (2016), who discovered that the addition of colostrum to yogurt increased the values of redness (a*) and yellowness (b*) while decreasing the values of lightness (L*) in all fortified yogurt samples. Storing time resulted in the yellowness of the cheese (b* values) being not significantly different (*P* > 0.05). Similar reports were given by Das et al. (2013), who reported no significant change in L*, a*, and b* values till the 12th day for dahi fortified with colostrum. The hue angle (H*), chroma (C*), Yellowing index (YI), and browning index (BI) were found to be improved in all fortified cheeses compared to the control group. Additionally, the improvement in color of the cheeses increased with higher percentages of added colostrum, indicating a positive correlation between colostrum concentration and quality enhancement. Similar results were observed during the storage period. These findings are consistent with those reported by El-Bialy et al. (2020), who reported an increase in saturation index (chroma) and Hunter hue angle in milk samples.

### Texture features

Cheese's texture is a crucial aspect of its quality. It is a mixture of various characteristics, including hardness, cohesiveness, gumminess, springiness, and chewiness. Gumminess is the product of both hardness and cohesiveness. Chewiness is the sum of both gumminess and springiness, which is the degree of flexibility at which cheese can be stretched before returning to its original length. The textural features of the fresh Cream cottage cheese were presented in Table S1. The highest value of hardness, cohesiveness, and gumminess was found for treatment T3, followed by T2 and T1, and the lowest value was found for the control sample. Generally, it was noticed that the raised level of the additional BCP increased the previous items, which could be explained by the reduced level of moisture and increased total solids in treatment cheeses compared with the control. The high protein and fat content of colostrum powder may account for the high viscosity of yogurt (Ayar et al. 2016) and the firmness of acid curd (Das and Seth 2017). The hardness can be an effective indicator of spreadability as well as resistance to biting and chewing. Total solids content influences hardness, emphasizing the importance of protein, protein-water interactions, and fat concentration in the formation of cheese product structure (Herrero and Requena 2005). The results of this study are in accordance with Koca and Metin (2004), who reported that dry matter influences the cohesiveness of cheese samples and that there is a direct relationship between dry matter and cohesiveness. Gumminess is the parameter that is the combination of both hardness and cohesiveness, so the factors that affect the hardness and cohesiveness affect the gumminess cumulatively. Moisture percentage, fat level, the structure of the protein matrix, and quantity of dry matter largely affect the gumminess of cheeses. Cohesiveness largely depends on the chemical composition of cheese. Herrera-Chávez et al. (2022) state that by adding colostrum to the cheese-making process, the protein content and yield of the cheese can be increased, and the dry matter can also be increased, resulting in greater cohesiveness of the cheese. The data also revealed that control cheeses have the highest values of springiness and chewiness compared to treatment cheeses. The decrease in elasticity of the protein matrix is influenced by fat that is present in the structure of a protein that is causing a decrease in the springiness of cheese (Delgado et al. 2011). Therefore, colostrum can increase cheese quality, especially in its physical characteristics. Hence, the higher level of casein in colostrum would improve the cheese's texture. It is expected to produce better-quality cheese as an innovative cheese product.

### Microbiological analysis of cream cottage cheese

The results in Table 5 illustrate the total bacterial, lactic acid bacteria, yeasts & molds, and coliform counts of Cream cottage cheese enriched by bovine colostrum powder during storage at 5 °C for 21 days. The total bacterial count of cheese fortified with different levels of BCP was significantly (*P* < 0.05) higher than the control on fresh and throughout the storage periods (7th, 14th, and 21st days) without negative effect on the quality of produced cheese during these storage period. This could be attributed to colostrum's higher micronutrient content, such as minerals and vitamins, which boosted the growth and activity of microorganism cultures (McGrath et al. 2016). During all storage periods, the counts of lactic acid bacteria and Bifidobacteria probiotics in cheese fortified with different levels of BCP were significantly higher than the control. Although colostrum contains antimicrobial substances (immunoglobulins, lactoperoxidase, lactoferrin, and lysozyme), this study showed that colostrum doesn’t have an adverse effect on the specific microbial flora of cheese. The examined cream cottage cheeses contained over 10^6^ CFU per gram of lactic acid bacteria, or Bifidobacteria, thus satisfying the criteria for a probiotic food product. Furthermore, the addition of colostrum promotes the growth of lactic acid bacteria by supplying a large amount of easily hydrolyzed (unfolded) proteins (Bomba et al. 2019). Furthermore, the lactic acid bacteria count in the control and all other treatments increased with storage time. Molds and yeasts were absent either when fresh or during the storage periods of up to 21 days in all treatments, which may be due to the presence of immune factors found in colostrum that block microorganism growth (Das et al. 2013) as well as, probably, the severity of heat treatments of milk and the preventive action of lactic acid bacteria. The coliform count for fresh C, T1 and T2 samples was below 10 CFU/g, but it was not present in the remaining storage period. In the case of T3, coliforms were not detected in both fresh samples and throughout the entire storage period. The overall pattern of these findings is consistent with the results presented by Das and Seth (2017), who observed the absence of mold & yeast, and coliforms in curd samples that were incorporated with colostrum powder.

**Table 5:** Effect of enrichment with colostrum powder on microbial quality of the resultant Cream cottage cheese during storage

Cream cottage cheese	Storage period (day)			
	1	7	14	21
*Total bacteria count (Log CFU /g****)***				
*C*	7.85 ± 0.07 ^aC^	7.91 ± 0.04 ^aC^	8.31 ± 0.08 ^cB^	9.13 ± 0.03 ^bA^
*T1*	7.42 ± 0.06 ^aD^	7.93 ± 0.02 ^aC^	8.49 ± 0.09b ^cB^	9.32 ± 0.02a ^bA^
*T2*	7.86 ± 0.07 ^aC^	7.89 ± 0.05 ^aC^	8.60 ± 0.10 ^bB^	9.86 ± 0.60 ^aA^
*T3*	7.45 ± 0.46 ^aC^	7.95 ± 0.02 ^aB^	9.30 ± 0.14 ^aA^	9.56 ± 0.06 ^abA^
*Lactic acid bacteria (Log CFU /g)*				
*C*	5.53 ± 0.01 ^cB^	5.81 ± 0.65 ^aAB^	6.32 ± 0.06 ^cA^	6.38 ± 0.05 ^bA^
*T1*	6.72 ± 0.08 ^aB^	6.72 ± 0.05 ^aB^	6.84 ± 0.02b ^AB^	6.90 ± 0.08 ^aA^
*T2*	6.90 ± 0.08 ^aA^	6.91 ± 0.01 ^aA^	6.92 ± 0.05 ^aA^	6.93 ± 0.05 ^aA^
*T3*	6.37 ± 0.28 ^bB^	7.04 ± 0.09 ^bA^	6.98 ± 0.00 ^aA^	6.95 ± 0.02 ^aA^
*Bifidobacteria ssp (Log CFU /g)*				
*C*	5.28 ± 0.55 ^bB^	5.33 ± 0.21 ^cB^	6.05 ± 0.06 ^cA^	6.11 ± 0.07 ^cA^
*T1*	5.69 ± 0.05 ^bC^	6.41 ± 0.09 ^aB^	6.61 ± 0.07 ^bA^	6.74 ± 0.08 ^abA^
*T2*	5.64 ± 0.12 ^bC^	5.80 ± 0.05 ^bB^	6.73 ± 0.07 ^abA^	6.70 ± 0.03 ^bA^
*T3*	6.56 ± 0.19 ^aA^	6.72 ± 0.31 ^aA^	6.79 ± 0.06 ^aA^	6.85 ± 0.06 ^aA^
*Mold & Yeast (Log CFU /g)*				
*C*	ND	ND	ND	ND
*T1*	ND	ND	ND	ND
*T2*	ND	ND	ND	ND
*T3*	ND	ND	ND	ND
*Coliform group (CFU/g)*				
*Control*	< 10 ^aA^	ND	ND	ND
*T1*	< 10 ^bA^	ND	ND	ND
*T2*	< 10 ^cA^	ND	ND	ND
*T3*	ND	ND	ND	ND

Mean (± SD) with small letters (abc) indicate significant differences (*P* < 0.05) among Cream cottage cheese samples enriched with different colostrum powder levels. Mean (± SD) with capital letters (ABC) indicate significant differences *P* < 0.05) among Cream cottage cheese samples during storage,where: C, control Cream cottage cheese; T1, Cream cottage cheese with 1% colostrum powder; T2, Cream cottage cheese with 2% colostrum powder; T3, Cream cottage cheese with 3% colostrum powder

### Organoleptic characteristics

The organoleptic scores of Cream cottage fortified with bovine colostrum powder during storage at 5 °C for 21 days are shown in Table 6. All organoleptic characteristics of cheese, such as colour and appearance, body and texture, and overall acceptability, had slightly increased scores, except flavor, which did not differ significantly and gained acceptance scores such as control. Colostrum powder fortification resulted in increased protein content, which might have improved the appearance and texture of cheese due to the interaction of whey proteins with casein micelles (Herrero and Requena 2005). The results showed that by adding colostrum to the cream cottage cheese, up to 2% of all organoleptic properties remained stable until the end of the storage period, but those of the control cheese had dropped from the 14th day to the end of storage. These results are in agreement with Das and Seth (2017), who indicated that the scores of body and texture, color, appearance, and overall acceptability of curd samples fortified with colostrum whey powder were significantly higher than the control, while the flavor scores did not change significantly when compared to the control. Similar reports were given by Nazir et al. (2018), wherein they reported that the product samples prepared from colostrum alone showed the highest mean scores for all the sensory characteristics, while the lowest mean scores were obtained by the product prepared from equal proportions of colostrum and whole milk. A comparison of the sensory attributes of the control and treated cheese (FPC) showed that it had a higher score for all sensory attributes than the control. This may be because colostrum cheeses had a soft and silky texture and a nutty flavor, while control cheese had a rough and coarse body and chewy texture, and that's due to the higher level of casein in colostrum would improve the cheese texture. Analysis of the data revealed that the ratio of colostrum addition and the storage period had a significant effect (*P* < 0.05) on the sensory attributes of cheese. At the end of the storage period, the scoring of all samples decreased due to the increase in acidity development. This could have been caused by the development of acidity or the production of microbial metabolism, which slightly harmed the product's rheological and sensory properties.

**Table 6:** Effect of enrichment with colostrum powder on organoleptic characteristics of the resultant Cream cottage cheese during storage

Cream Cottage cheese	Storage period (day)	Flavor (1–10)	Body and texture (1–5)	Appearance and color (1–5)	Overall acceptability (1–20)
C	1	9.58 ± 0.66^aA^	4.08 ± 0.38 ^cA^	4.50 ± 0.45^b A^	18.17 ± 0.88^aA^
	7	9.58 ± 0.66 ^aA^	4.04 ± 0.33^cA^	4.63 ± 0.38^abA^	18.25 ± 0.97^aA^
	14	9.04 ± 0.87 ^aAB^	4.00 ± 0.45^bA^	4.50 ± 0.00^aA^	17.54 ± 1.23 ^aAB^
	21	8.33 ± 0.82 ^aB^	3.50 ± 0.00^bB^	4.17 ± 0.61^aA^	16.00 ± 0.84^aB^
T1	1	9.08 ± 0.49 ^aA^	4.50 ± 0.45^bA^	4.92 ± 0.20 ^aA^	18.50 ± 0.95^aA^
	7	9.08 ± 0.49 ^aA^	4.50 ± 0.45^bA^	4.92 ± 0.20^aA^	18.50 ± 0.95^aA^
	14	8.67 ± 0.41 ^aAB^	4.42 ± 0.20 ^aA^	4.50 ± 0.00^aB^	17.58 ± 0.49^aAB^
	21	8.25 ± 0.42^aB^	4.25 ± 0.27 ^aA^	4.42 ± 0.20^aB^	16.92 ± 0.58^aB^
T2	1	9.25 ± 0.42 ^aA^	4.83 ± 0.26 a ^bA^	4.67 ± 0.26^abA^	18.75 ± 0.52^aA^
	7	9.17 ± 0.61 ^aA^	4.81 ± 0.25^abA^	4.67 ± 0.26^abA^	18.64 ± 0.72^aA^
	14	8.75 ± 0.27^aAB^	4.58 ± 0.20^aAB^	4.58 ± 0.20^aA^	17.92 ± 0.38^aB^
	21	8.58 ± 0.38^aB^	4.42 ± 0.20^aB^	4.42 ± 0.20^aA^	17.42 ± 0.58^aB^
T3	1	8.92 ± 0.49^aA^	5.00 ± 0.00^aA^	4.42 ± 0.20^bA^	18.33 ± 0.41^aA^
	7	8.92 ± 0.49 ^aA^	5.00 ± 0.00^aA^	4.42 ± 0.20 ^bA^	18.33 ± 0.41^aA^
	14	8.83 ± 0.52^aA^	4.58 ± 0.38^aB^	4.42 ± 0.20 ^aA^	17.83 ± 0.41^aB^
	21	8.50 ± 0.32 ^aA^	4.33 ± 0.41^aB^	4.33 ± 0.41^aA^	17.17 ± 0.26^aC^

Mean (± SD) with small letters (abc) indicate significant differences (*P* < 0.05) among Cream cottage cheese samples enriched with different colostrum powder levels. Mean (± SD) with capital letters (ABC) indicate significant differences *P* < 0.05) among Cream cottage cheese samples during storage,where: C, control Cream cottage cheese; T1, Cream cottage cheese with 1% colostrum powder; T2, Cream cottage cheese with 2% colostrum powder; T3, Cream cottage cheese with 3% colostrum powder

Therefore, based on both the results obtained and the sensory evaluation of the product, it can be concluded that the concentration of 2% is the most effective concentration in terms of influencing the characteristics of the resulting cottage cheese and its susceptibility.

## Conclusions

Despite its numerous immunological benefits, colostrum is still an underutilized food in the dairy industry due to consumer aversion to it and its high perishability. And its usage, or that of any of its constituents, is currently quite limited in dairy foods. However, due to its high protein and bioactive component content, it can be used to fortify dairy products. From the present study, it could be concluded that the enrichment of cottage cheese by the addition of bovine colostrum particularly, with 2% and with probiotic bacteria will increase the nutritional value and health benefits of cheese and thus enhance the quality and overall acceptability of the product, allowing it to be consumed as a new functional dairy food suitable for all categories of people, from babies to adults.

### Supplementary Information

Below is the link to the electronic supplementary material.

Supplementary file1 (DOCX 28 kb)

## Data Availability

The authors confirm that the data supporting the findings of this study are available within the article and its supplementary materials.
