# Supplementary material for: Production and evaluation of novel functional cream cottage cheese fortified with bovine colostrum and probiotic bacteria
Source: J Food Sci Technol. 2024 Jan 19;61(8):1457–69. doi: 10.1007/s13197-023-05910-0 (PMC11219591; doi:10.1007/s13197-023-05910-0)
Supplement: Supplementary file 1 — Supplementary file1 (DOCX 28 kb) [file 13197_2023_5910_MOESM1_ESM.docx]

Table S1: Effect of enrichment with colostrum powder on Texture properties of the resultant fresh Cream cottage cheese

| Texture properties | Cream Cottage cheese | | | |
| --- | --- | --- | --- | --- |
|  | C | T1 | T2 | T3 |
| Hardness (N) | 5.30±0.27^b^ | 5.60±0.28^b^ | 6.60±0.33^a^ | 6.80±0.34^a^ |
| Cohesiveness (B/A area) | 0.73±0.04^a^ | 0.73±0.04^a^ | 0.75±0.04^a^ | 0.80±0.04^a^ |
| Springiness | 0.90±0.05^a^ | 0.88±0.04^a^ | 0.81±0.04^a^ | 0.81±0.04^a^ |
| Gumminess | 4.10±0.21^b^ | 4.20±0.21^b^ | 4.50±0.23^a^ | 4.93±0.15^a^ |
| Chewiness | 3.86±0.19^a^ | 3.24±0.16^a^ | 3.20±0.16^a^ | 2.17±0.11^b^ |

Means (three different determinations) ± standard deviation (SD)

**Fig. S1 Fatty acid profile of colostrum powder (g/100 g total fatty acid)**
